# Avapritinib reduces symptoms and mast cell burden in systemic mastocytosis

**DOI:** 10.1186/s13223-025-00986-z

**Published:** 2025-09-17

**Authors:** Paula Nöldeke, Oliver Schmalz, Hans Kvasnicka, Jens Panse, Silke C. Hofmann

**Affiliations:** 1 Center for Dermatology, Allergology and Dermatosurgery, Helios University Hospital Wuppertal, University Witten/Herdecke, Heusnerstr. 40, 42283 Wuppertal, Germany; 2 Department for Hematology, Oncology and Palliative Medicine, Helios University Hospital Wuppertal, University Witten/Herdecke, Wuppertal, Germany; 3 Institute of Pathology, Helios University Hospital Wuppertal, University Witten/Herdecke, Wuppertal, Germany; 4 Center for Integrated Oncology Aachen Bonn Cologne Düsseldorf (CIO ABCD), Aachen, Germany; 5 Department of Hematology, Oncology, Hemostaseology and Stem Cell Transplantation, University Hospital RWTH Aachen, Aachen, Germany

**Keywords:** Mast cell, Osteoporosis, PROMs, Tryptase

## Abstract

**Background:**

Mastocytosis is driven by a clonal expansion of mast cells, commonly triggered by the KIT D816V mutation which is present in over 90% of adult patients. Individuals with indolent systemic mastocytosis (ISM) frequently experience recurrent anaphylaxis and mast cell mediator-related symptoms, leading to substantial morbidity. In rare cases, progression to more severe subtypes, such as smoldering systemic mastocytosis (SSM), can occur.

**Case presentation:**

We describe one patient with ISM and another with ISM transitioning to SSM, both treated with the selective KIT D816V inhibitor avapritinib at a daily dose of 25 mg. Following initiation of avapritinib, both patients exhibited a marked reduction in serum tryptase levels and complete remission of maculopapular cutaneous mastocytosis. Additionally, joint pain, gastrointestinal symptoms, and neurocognitive complaints decreased. Sustained clinical improvement over follow-up periods of 9 and 12 months was consistently reflected in disease-specific patient-reported outcome measures (PROMs).

**Conclusions:**

Regular clinical and laboratory monitoring, including serum tryptase and KIT D816V mutation assessment in peripheral blood, is essential in all ISM patients to detect early signs of disease progression. In refractory cases, avapritinib is a promising therapeutic option that can reduce mast cell burden, alleviate symptoms, and enhance overall quality of life.

## Introduction

Mastocytosis is caused by a clonal expansion of mast cells, most commonly triggered by the KIT D816V mutation present in over 90% of adult patients [1]. While aggressive forms of systemic mastocytosis (SM) carry a poor prognosis, non-advanced SM significantly impairs quality of life due to an increased risk of recurrent anaphylactic episodes and/or mast cell mediator-related symptoms affecting the skin, nervous system, musculoskeletal system, and gastrointestinal tract [2, 3].

The diagnosis and proper classification of SM rely on bone marrow smears and/or biopsy findings. According to the WHO 5th edition classification, multifocal dense infiltrates of mast cells are required as a major criterion along with at least one minor criterion, or presence of at least 3 minor criteria: ≥ 25% atypical mast cells, mast cells with aberrant expression of CD25, CD30, and/or CD2, presence of the KIT D816V mutation, or a baseline serum tryptase level > 20 µg/l [4].

The current WHO classification comprises three types of non-advanced SM: indolent systemic mastocytosis (ISM) with or without maculopapular cutaneous mastocytosis (MPCM), bone marrow mastocytosis without skin changes, and the rare smoldering systemic mastocytosis (SSM). The latter is characterized by presence of at least 2 B-findings indicating a particularly high mast cell burden with signs of myeloproliferation, myelodysplasia, or organomegaly not impairing organ function [4]. In detail, a variant allele frequency ≥ 10% for KIT D816V, an infiltration grade of ≥ 30% mast cells in histology, or serum tryptase levels ≥ 200 µg/l suggest a high mast cell burden.

Treatment goals for non-advanced SM patients are control of symptoms with H1-/ H2-antihistamines and mast cell stabilizers, prevention of anaphylaxis, and treatment of osteoporosis [2]. Multikinase inhibitors (e.g. midostaurin) are employed in aggressive SM, but carry a risk for cytopenias and increased infections. Avapritinib is a selective KIT D816V inhibitor which has been approved in Europe since December 2023 for ISM defined by WHO criteria with moderate to severe symptoms refractory to symptomatic therapy [5]. Previously, avapritinib was approved for advanced SM following randomized trials that demonstrated improved overall survival, a reduction in bone marrow mast cell burden and tryptase levels, and substantial alleviation of symptoms [6, 7].

We present one patient with ISM and another patient with ISM in transition to SSM, who experienced clearance of MPCM, symptomatic relief, and a decrease in tryptase and KIT D816V mutational load demonstrating a disease modifying effect of low-dose avapritinib with excellent tolerability. Improvement of signs and symptoms of SM was measured and monitored in both patients using disease-specific, valid and reliable patient-reported outcome measures (PROMs).

## Case report

### Case 1

**Fig. 1:**
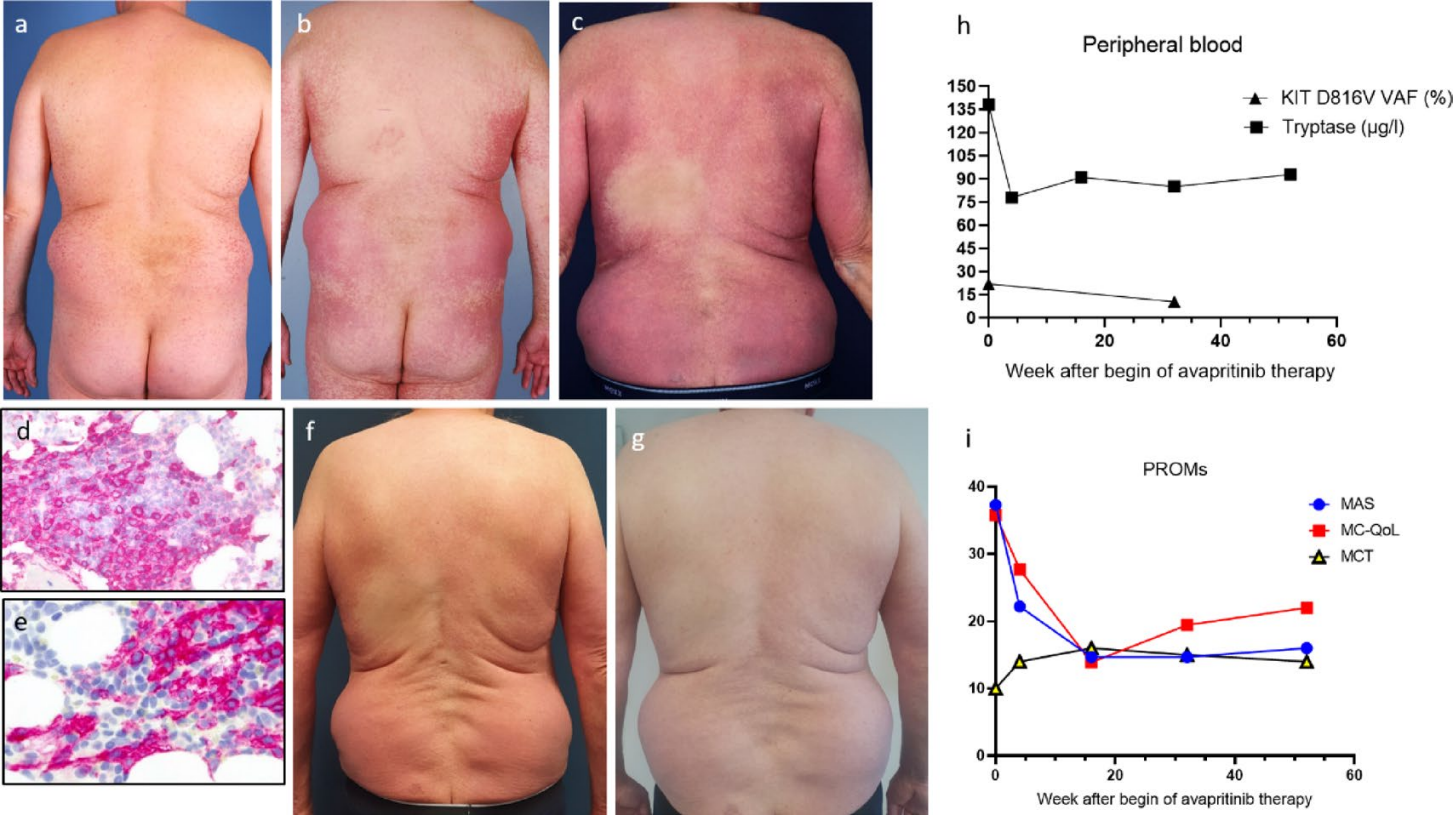
Patient 1: Moderately pronounced erythematous macules on the trunk at first presentation in 2005 **a** became increasingly livid and confluent within the following 9 years **b** resulting in a disseminated and severe MPCM before initiation of avapritinib in 2024 (**c**). Bone marrow biopsy showed dense tryptase-positive atypical mast cell infiltrates (**d**), with > 25% of the cells appearing spindle-shaped in a CD117 staining (**e**). Two months after initiation of avapritinib, the skin lesions appeared lighter (**f**), and after one year, they were barely recognizable (**g**). The decrease in mast cell burden was reflected by a significant decrease in serum tryptase and the variant allele frequency for KIT D816V in peripheral blood (**h**). Symptomatic improvement was paralleled by a decrease in the mastocytosis activity score (MAS) and quality of life score (MC-QoL), and an increase in the mastocytosis control test (MCT) (i)

A 48-year-old man first presented in 2005 with flushing, pruritus, occasional diarrhea, elevated serum tryptase of 44 µg/l (norm < 11), and erythematous macules on trunk and thighs (Fig. 1a). While MPCM was confirmed histologically, further diagnostics including a marrow biopsy, gastroscopy, colonoscopy and imaging procedures, revealed no abnormalities. High-dose UVA-1 therapy led to a slight and temporary improvement in MPCM. Overall, the clinical course remained stable over several years with symptomatic therapy consisting of antihistamines (desloratadine, fexofenadine) and oral cromolyn sodium.

In 2014 the patient complained of frequent gastrointestinal symptoms, pronounced itching after physical activity or exposure to heat, and cognitive impairment (brain fog, difficulty concentrating, fatigue). His tryptase level had risen to 84.2 µg/l. A repeat bone marrow biopsy showed multifocal infiltration with CD117 and CD25 positive atypical mast cells (approximately 5% of all hematopoietic cells) and evidence of the KIT D816V mutation leading to the diagnosis of ISM. In addition, colonoscopy revealed clustered CD117 and CD25 positive mast cells indicating intestinal mastocytosis. Mild osteoporosis was detected by dual-energy X-ray absorptiometry (DEXA) and subsequently treated with vitamin D.

In the following years, MPCM, pruritus, urticaria, and heat intolerance were worsening, the gastrointestinal complaints and fatigue persisted, and the tryptase level increased up to 138 µg/l. A further marrow biopsy revealed atypical spindle-shaped clustered mast cells according to the WHO major criterion with an infiltration level of up to 20% (Fig. 1b–e). In addition, new splenomegaly and a high variant allele frequency (VAF) of 22% for KIT D816V in peripheral blood represented two B-findings indicating a transition to SSM. A repeat DEXA scan showed a median T-score of -3.63, pointing to an increased risk of bone fractures.

Since the patient refused inclusion in the summit study for bezuclastinib, we initiated targeted mast cell-depleting therapy with avapritinib 25 mg/d orally in May 2024. Within 4 weeks MPCM, itch, flushing, and diarrhea improved significantly, while fatigue and bone pain were slightly regressive. The favourable course of the disease was reflected by PROMs: the Mastocytosis Activity Score (MAS) assesses 9 items including 3 skin-specific symptoms (itching, wheals, flushing), 2 gastrointestinal complaints (diarrhea, abdominal cramps) in addition to muscle or joint pain, fatigue, headache, and difficulty in concentrating [8]. Within 4 months the MAS fell from 37.3 to 15, and the health-related quality of life impairment measured by the 27-item Mastocytosis Quality of Life Questionnaire (MC-QoL) from 35.8 to 13.9 [9]. In contrast, the Mastocytosis Control Test (MCT) increased from 10 to 16 reflecting a higher level of disease control [10]. Splenomegaly resolved and serum tryptase dropped, but remained elevated over the past year at 90 µg/l. The KIT D816V VAF in peripheral blood, however, came down from 22.0 to 10.5%, indicating a disease-modifying efficacy of avapritinib (Fig. 1f–i). A duplication of the TPSAB1 gene was not detected in this patient, allowing us to rule out hereditary alpha-tryptasemia.

### Case 2

**Fig. 2:**
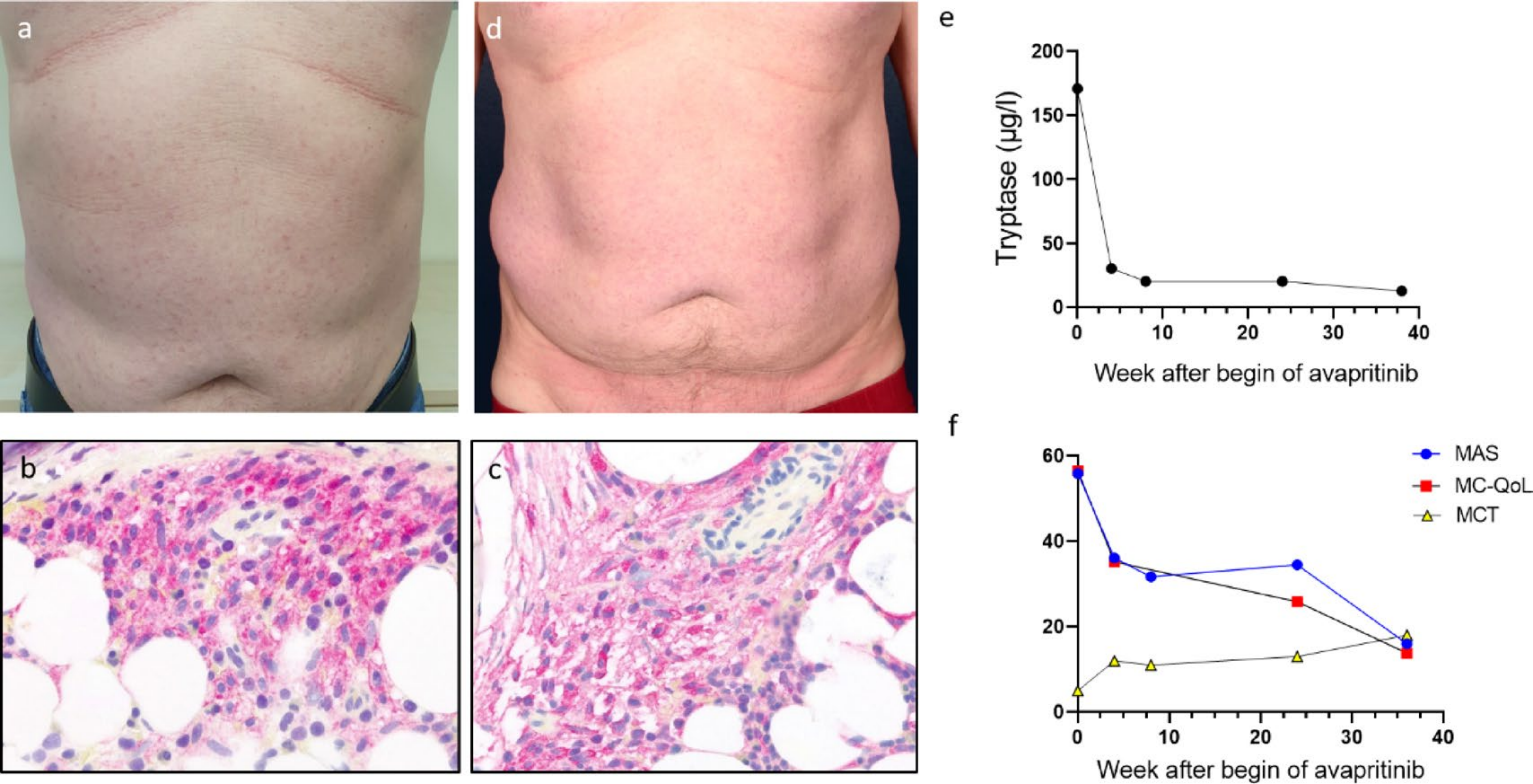
Patient 2: Before initiation of avapritinib, discrete MPCM on the trunk (**a**), and dense tryptase-positive mast cell infiltrates in the bone marrow (**b**) with co-expression of CD25 (**c**) were present consistent with the diagnosis of ISM. Within 2 months of avapritinib therapy, skin lesions had cleared (**d**). Serum tryptase decreased rapidly, reaching near-normal levels (13 µg/l) within 9 months (**e**). Furthermore, a continuous and sustained improvement in PROMs was observed (**f**)

A 58-year-old male patient first presented in 2022 with a history of MPCM for 15 years. The patient complained of itching and recurrent urticaria, especially after hot showers since 2 years. A diagnosis of ISM was made based on strongly elevated serum tryptase at 196 µg/l, and a marrow biopsy revealing CD25- and CD117-positive atypical, mainly spindle-shaped, clustered mast cells with an infiltration level of up to 20% and evidence of the KIT D816V mutation (Fig. 2a–c). Tryptase genotyping excluded a diagnosis of hereditary alpha-tryptasemia.

In the following year, flushing, generalized pruritus, urticaria (> 50 wheals/day), fatigue, brain fog, headache, vertigo, and gastrointestinal complaints worsened despite symptomatic treatment with ketotifen, desloratadine (4 × 5 mg/d) and famotidine. A DEXA scan revealed a median T-score of − 2.75 suggesting early-stage osteoporosis, which was subsequently treated with vitamin D supplementation. KIT D816V was not detectable in peripheral blood, but in the bone marrow the VAF increased from 0.22% in 2022 to 2.6% in 2024.

Within 4 weeks after initiation of avapritinib 25 mg/d in October 2024, serum tryptase decreased to 30.6 µg/l, and cutaneous symptoms (MPCM, pruritus, and urticaria) similarly to fatigue, brain fog, nausea, and vertigo improved. Over the subsequent months, cutaneous, gastrointestinal, and neurocognitive symptoms continued to subside, as reflected by PROMs: within 9 months, the MAS fell from 55.9 to 16.0, the MC-QoL from 56.5 to 13.8, while the MCT rose from 5 to 18. Consistent with the sustained clinical improvement, serum tryptase levels remained below 20 µg/l (Fig. 1d–f).

## Discussion

Targeted therapies have revolutionized the management of numerous malignant diseases. Avapritinib is a highly selective, potent inhibitor of the D816V mutated KIT oncogene able to reduce serum tryptase and bone marrow mast cell counts by at least 50% in SM according to randomized placebo-controlled studies [5–7].

The various complaints of our patients illustrate the high disease burden and impairment in quality of life despite optimized symptomatic therapy with antihistamines and mast cell stabilizers. Both patients significantly benefited from avapritinib 25 mg daily reflected at first by an impressive and rapid improvement of flushing, itching, urticaria, and MPCM.

Similarly to our cases, Lee et al. demonstrated complete resolution of severe MPCM and a sustained decrease of serum tryptase levels with avapritinib given at 130 mg daily in a woman with aggressive SM. This was associated with a normalization of mast cell counts in repeated skin biopsies [11]. Furthermore, 100 mg avapritinib was previously shown to reduce pruritus, MPCM, brain fog, gastrointestinal symptoms, and flushing in a female patient with ISM after several lines of tyrosine kinase inhibitor therapy including imatinib and midostaurin [12].

The reduction of mast cell burden in the skin, with MPCM representing the most severe symptom burden after fatigue, was a major outcome of the original PIONEER trial assessing the efficacy and safety of low-dose avapritinib [5]. Furthermore, initial tryptase levels > 20 µg/l were associated with greater therapeutic benefits corresponding to the real-life efficacy of avapritinib in our patients [5].

The less significant reduction in serum tryptase in patient 1 compared to patient 2 during targeted therapy is notable and may reflect the higher baseline mast cell burden, as indicated by a KIT D816V VAF of 22% in peripheral blood versus 2.6% in bone marrow, respectively. In advanced SM, lower mutation burdens were observed to correlate with better response rates and faster molecular responses to avapritinib [7].

Improving the diverse symptoms and quality of life is a key goal of SM treatment [13]. The mastocytosis-specific validated PROMs MAS, MC-QoL, and MCT help to assess disease activity, quality of life, and disease control [8–10] in patients with ISM. Regular assessment with these PROMs during avapritinib therapy allowed us to demonstrate a sustained response to low-dose treatment in our patients.

Noteworthy, approximately 90% of patients with MPCM have an associated SM [14], although the diagnosis cannot always be confirmed initially. In our first patient, the erythematous macules gradually became livid and confluent over 19 years, a morphology and clinical course associated with an increased risk of progression to advanced SM [11]. B-findings (splenomegaly and a high KIT D816V VAF) were finally proven corresponding to evolving SSM [2, 4]. Thus, this case demonstrates the relevance of long-term clinical and laboratory monitoring including annual determination of serum tryptase, and repeated bone marrow biopsy, abdominal ultrasonography and DEXA scan every 3–5 years for early detection of disease progression [15].

Moreover, non-invasive determination of the KIT D816V allele burden in EDTA blood enables physicians to identify patients at risk [16] and to monitor mast cell depletion during targeted therapy.

## Conclusion

Although ISM and SSM represent non-advanced variants of SM, they are associated with high morbidity and a significantly decreased quality of life, often despite symptomatic therapy.

Our patients experienced a disease modifying effect with excellent tolerability of low-dose avapritinib as shown by clearing of cutaneous lesions, decrease in tryptase and mutational load, and improvement in neurocognitive, musculoskeletal, and gastrointestinal symptoms. These well-documented cases further support the rapid and durable efficacy of low-dose avapritinib in non-aggressive SM.

## Data Availability

No datasets were generated or analysed during the current study.

## Abbreviations

DEXA: Dual energy X-ray absorptiometry
MPCM: Maculopapular cutaneous mastocytosis
ISM: Indolent systemic mastocytosis
PROM: Patient-reported outcome measures
SM: Systemic mastocytosis
SSM: Smoldering systemic mastocytosis
